# Short-term transfer effects of Tetris on mental rotation: Review and registered report — A Bayesian approach

**DOI:** 10.3758/s13414-024-02855-0

**Published:** 2024-02-22

**Authors:** J. David Timm, Markus Huff, Stephan Schwan, Frank Papenmeier

**Affiliations:** 1 Department of Psychology, University of Tübingen, Schleichstr. 4, D-72076 Tübingen, Germany; 2 Leibniz-Institut für Wissensmedien (IWM) in Tübingen, Tübingen, Germany

**Keywords:** Transfer effect, Video games, Mental rotation, Tetris, Bayes

## Abstract

The existence of transfer effects of video games on cognitive performance are controversially discussed in experimental psychology. Whereas recent meta-analyses suggest the absence of far transfer effects, empirical evidence regarding near transfer effects is more controversial. This conceptual replication investigated the short-term near transfer effect of playing Tetris on mental rotation abilities. The design of the conceptual replication was based on a comprehensive compilation of the methods used by previous literature on this topic and advanced in order to reach a high scientific state-of-the-art standard. We ran a high-powered conceptual replication study with 366 participants randomly assigned to either an experimental group playing Tetris or a control group playing Solitaire. Both groups completed three commonly used mental rotation tests in a pre- and a posttest session. Additionally, the experimental group played Tetris while the control group played Solitaire. Playing time was 10 hours in total within 4 weeks. Based on previous research, we hypothesized that this might generate a short-term transfer effect of Tetris on mental rotation. While participants showed a repeated testing effect for the mental rotation tests in both groups, we found evidence that Tetris does not produce a short-term transfer effect on mental rotation. Both gender and expected outcomes did not influence this effect. Our study suggests that playing Tetris does not improve mental rotation skills.

## Background

Video games are among the most popular and frequent leisure-time activities in today’s culture. Since the beginning of video game usage, researchers have been interested in the cognitive effects of gaming. A generalized transfer effect of video games on cognitive performance is a controversially discussed topic in experimental psychology (Bediou et al., 2018). There is a wide range of research in this field with different findings, but recent meta-analyses proposed that there are no reliable far and long-term transfer effects of video games and that there are also contradictory findings regarding the existence of short-term near transfer effects of video game play (Meneghetti et al., 2016, 2018; Moreau, 2013; Quaiser-Pohl et al., 2006; Sala et al., 2018). With the present study, we want to contribute to this discussion by the publication of a data set that is unbiased by its statistical significance. Thus, we focused on and conducted a registered conceptual replication report of the short-term transfer effect of playing Tetris on mental rotation abilities, which is one of the most used and cited short-term near transfer effects (e.g., Boot et al., 2008; De Lisi & Wolford, 2002; Okagaki & Frensch, 1994). We investigated the theory of the specific transfer of general skills (as described in Pilegard & Mayer, 2018; Sims & Mayer, 2002), which states that learning a skill in a game will transfer to performance on the same skill in another venue outside the same.

In order to ensure a comprehensible conceptual replication, we searched with the keywords Tetris, training, game, mental rotation, transfer and/or effect in PsycARTICLES, PsycINFO, PSYNDEX, and grey literature. Twenty-seven studies were included in the first step. In the second step, we excluded all studies that did not directly relate Tetris to mental rotation, did not have a control group, did not manipulate solely training of Tetris or that did not specify sample characteristics or experimental procedure. Thus, we finally included eight studies into the present review and into the conceptual replication. This research used at least one of three common mental rotation tests (see Table 1) and was published within the period from 1994 to 2018 (see Table 2). Taking a closer look into the literature, however, the measured variables and effects differ heavily across studies.

**Table 1:** Common mental rotation tests

Test	Dimension	Items	Maximum points	Task
Card Rotations Test (CRT)	2D	20	160 (80^1^) (39^2^)	Participants must detect whether a rotated card is the same as or different from the reference shape (referred to as card). Each item has a reference card on the left and eight test cards on the right. Participants must choose for every card whether it is the same or a different card compared with their reference card. Participants have 6 minutes for this test. One point per card is given. If one card is interpreted falsely, a negative point is given and subtracted from the general score (Ekstrom et al., 1976)
Cube Comparisons Test (CCT)	3D	42	42	Participants must detect whether a rotated cube is the same as or different from the reference shape (referred to as cube). Each item has a reference cube on the left and a test cube on the right. Participants must choose for the test cube whether it is the same or a different cube compared with their reference cube. Participants have 6 minutes for this test. One point per cube is given. If one cube is interpreted falsely, a negative point is given and subtracted from the general score (Ekstrom et al., 1976).
Mental Rotation Tests (MRT)	3D	20/24	20/24 (40^3^)	Participants see a Tetris-block-like reference shape and must detect the two rotated versions from four alternatives. Participants have 6 minutes for this test. For each item, one point was given if participants correctly indicated the two rotated alternatives and no false alternative. There are no negative points (Peters et al., 1995; Vandenberg & Kuse, 1978).

^1^Sims and Mayer (2002); Pilegard and Mayer (2018);^**2**^ De Lisi and Wolford (2002); ^**3**^ De Lisi and Cammarano (1996)

**Table 2:** Studies investigating short-term transfer of Tetris on mental rotation characteristics (ordered chronologically)

Study	Mental Rotation Test*	Learning Outcomes Reported	Transfer Effects (TE) by Gender	Sample Size	Training Duration	Control Condition (CC)
Okagaki and Frensch (1994)	CRT, CCT, & MRT (modified)	Playing Tetris improved mental rotation skills (CCT & MRT only)	Mixed findings	*N* = 57	6 h (12 × 0.5 h, within 2 weeks)	Nonactive CC
De Lisi and Cammarano (1996)	MRT	Playing Tetris improved mental rotation skills	Stronger TE for males	*N* = 110	1 h (2 × 0.5 h, 1 week between)	CC played Solitaire (strategic card video game)
Sims and Mayer (2002)	CRT & MRT (modified)	Playing Tetris did not improve mental rotation skills compared with a matched control group	Female participants only	*N* = 16	12.3 h (1 × 20 min + 12 × 1 h, within 4 weeks)	Nonactive CC
De Lisi and Wolford (2002)	CRT (modified)	Playing Tetris improved mental rotation skills	Mixed findings	*N* = 47	5.5 h (11 × 0.5 h, within 4 weeks)	CC played Carmen Sandiego (educational mystery video game)
Cherney (2008)	CRT & MRT	Playing Tetris improved mental rotation skills	Stronger TE for females	*N* = 61	4 h (0.5 h + 3 × 1 h + 0.5 h, within 1 or 2 weeks)	CC solved Puzzle (real world)
Terlecki et al. (2008)	MRT	Playing Tetris did not improve mental rotation skills compared with a matched control group	Mixed findings	*N* = 180	12 h (12 × 1 h, within 12 weeks)	CC played Solitaire (strategic card video game)
Boot et al. (2008)	MRT (modified)	Playing Tetris improved mental rotation skills	Not reported	*N* = 82	21.5 h (session duration not reported, 4–5 weeks)	CC played either Medal of Honor, Rise of Nations, or was nonactive
Pilegard and Mayer (2018)	CRT	Playing Tetris did not improve mental rotation skills compared with a matched control group	Not reported	*N* = 66 (*n* = 49: 2 Tetris groups)	4 h (4 × 1 h + pretest, within 5 weeks)	Nonactive CC

*The column Mental Rotation Test includes only standardized mental rotation tests used in the respective study

In the following three paragraphs, we present the mental rotation tests used including the transfer effects found in comparison, varying gender effects across studies, as well as experimental procedures. One major difference between studies in this field is the measurement of mental rotation abilities. Three different tests are used commonly (see Table 1), but different patterns were found with these tests. In one of the first studies investigating the effect of Tetris on mental rotation, playing Tetris improved reaction time regarding mental rotation and led to higher scores on the CCT (Okagaki & Frensch, 1994). For the CRT, they did not observe any training effect that was specific to Tetris (Okagaki & Frensch, 1994). However, 8-to-9-year-old children improved their mental rotation skills on the CRT if they performed below average before (De Lisi & Wolford, 2002).

Cherney (2008) reported improved CRT scores caused by Tetris. However, this study failed to report the interaction with the game played (Tetris vs. control). Instead, the control group also showed a significant improvement in CRT, leaving open the possibility that there was only a repeated testing improvement for the CRT instead of a training effect that was specific to Tetris. Sims and Mayer (2002) found no specific transfer effect of Tetris on the CRT, but found a repeated testing effect as well. Using the MRT, small to moderate short-term transfer effects of Tetris were reported (De Lisi & Cammarano, 1996; Terlecki et al., 2008). However, the transfer of Tetris and action video games regarding mental rotation seem to be comparable (Cherney, 2008). Furthermore, it seems surprising that participants improved more on 3D measures (MRT) than on 2D measures (CRT) of mental rotation, considering that Tetris trains 2D rotations rather than 3D rotations. To summarize, these mental rotation tests show different outcomes in different experimental sets, and this might be a first hint that short-term near transfer effects of Tetris on mental rotation are not as robust as sometimes described (see Table 2).

These eight studies do not differ only with regard to the reported transfer effects for different measures of mental rotation abilities. There are also varying gender effects (see Table 2). These gender differences across the mental rotation tests reported are not consistent, sometimes favoring a better transfer effect in males (De Lisi & Cammarano, 1996; Okagaki & Frensch, 1994) and sometimes the other way round (Cherney, 2008). This might provide another indication that short-term transfer effects of Tetris on mental rotation are not as robust as sometimes reported.

Another major difference across these studies is the experimental procedure including the training duration, sample size and control condition as shown in Table 2. Sample size ranged from 16 to 180 across studies. Within eight studies, there were five different control group conditions. Only Solitaire (De Lisi & Cammarano, 1996; Terlecki et al., 2008) and not playing any game (Okagaki & Frensch, 1994; Pilegard & Mayer, 2018; Sims & Mayer, 2002) were used more than once. Furthermore, the duration of both the whole experiment (1 to 12 weeks) and individual sessions (20 to 60 minutes) differed a lot.

All in all, the results and procedures of the studies reported deliver contradictory conclusions. As there are various methodological variations across the studies reported, we performed a conceptual replication based on a comprehensive compilation of the methods used by previous literature rather than a precise replication of a particular study in order to strike a balance between the different studies, which found evidence either for or against a short-term transfer effect induced by practicing Tetris.

The replication of the effect of Tetris on mental rotation abilities is not only interesting in itself. Rather, it has a strong impact on the debate about the existence of short-term game transfer effects in general because it is one of the nearest transfer effects in gaming literature. If this effect cannot be replicated consistently, then other near game transfer effects may also need to be questioned regarding their reproducibility. There are many advantages of this registered replication, which can have a strong impact on future research. First, this study is highly powered, unbiased by statistical significance and was peer reviewed prior to the publication in order to maximize objectivity. Second, we used the Bayesian statistics framework. Thus, we can draw a conclusion about a transfer effect independently of the presence or absence of the effect, in detail we are able make a decision favoring the null or the alternative hypothesis, which stands in contrast to all former published NHST papers in this field. Third, we give a summary about previous research regarding this topic and provide differences and commonalities, which result in a well-considered method. In accordance with the AP&P RRR guide, we state that there are no previous, related experiments, published or unpublished, conducted by the authors regarding this subject.

## Methods

We matched the methods in the light of consensus in the field — namely, an efficacy study as proposed by Green and colleagues (2019).

## Participants

Given the huge variability in effect sizes in literature (Boot et al., 2008; Cherney, 2008; De Lisi & Cammarano, 1996; De Lisi & Wolford, 2002; Okagaki & Frensch, 1994; Pilegard & Mayer, 2018; Sims & Mayer, 2002; Terlecki et al., 2008), we calculated a Bayes factor design analysis (BFDA) for a sequential design and two-sided Bayesian *t* test assuming a medium effect size of *d* = 0.5, a prior as zero-centered Cauchy distribution with a scale parameter of $\sqrt{2}/2$, asymmetric Bayes factor boundaries of 1/6 (≈ 0.167) and 10, and a minimum sample size of 50 participants per group (Schönbrodt & Wagenmakers, 2018). In order to achieve a conclusion rate of .8, we would need to collect 176 participants per group under the assumption that the H0 is true and 106 participants per group under the assumption that the H1 is true. Following our BFDA, we decided to recruit a minimum of 50 participants per group and a maximum of 176 participants per group and to apply an optional stopping rule once any of the two Bayes factor boundaries are reached for each of the mental rotation tests investigated while the balance across conditions is not violated. This left us with a probability of .968 for true positive evidence, .002 for false negative evidence, and .03 for inconclusive evidence under the assumption of a true effect of 0.5 and a probability of .788 for true negative evidence, .017 for false positive evidence, and .196 for inconclusive evidence under the assumption of no effect. Following these rules, we collected the data of 366 participants (males and females were equally distributed across groups). Participants were paid 5€ for the first session and 5€ for the last session while the playing time was promoted with a lottery drawing of shopping vouchers (10 × 100€ and 20 × 50€). The BFDA script and the simulated data can be found online (https://osf.io/d3wuc/).

## Materials

The game used in the experimental group was Tetris. The control group played Solitaire. We measured mental rotation abilities with the three commonly used rotation tests, in detail CRT, CCT, and MRT (see Table 1). This study was conducted online. Therefore, we presented electronic versions of the mental rotations test. Furthermore, we measured participants’ expectations regarding the influence of playing Tetris and Solitaire on mental rotation performance (Boot et al., 2013).^1^ We measured expectations before game exposure in order to avoid confounding factors due to the manipulations.

## Procedure

The procedure applied was based on a comprehensive compilation of the methods used by previous literature rather than a precise replication of a particular study in order to strike a balance between the different studies, which found evidence either for or against a short-term transfer effect induced by practicing Tetris. Mental rotation tests were taken twice, as pre- and posttest. The order of the three mental rotation tests was balanced across participants and applied to pre- and posttest. In the beginning, we measured the expected outcomes within a masked questionnaire including questions about the effect of Tetris and Solitaire on mental rotation abilities. After completing the pretest, participants were asked to play for 10 hours in a self-paced manner within a maximum of 4 weeks and a maximum playing time of 2 hours per day (actual playing times logged by our online platform to ensure the intended practice times). The experimental group played Tetris and the control group played Solitaire. After 10 hours of game practice, the posttest was taken.

## Analysis

In order to ensure that all participants entering our analysis were engaged in Tetris, we replaced any participant that played less than 10 hours within 4 weeks. We increased logged playing time only whenever there was an activity every 15 seconds for Tetris and 60 seconds for Solitaire. We first computed the pre–post difference score for each mental rotation test and participant. The pre–post difference was calculated to control for repeated testing effects. Thus, we can interpret the improvements regarding video game practice. Thereafter, we compared those difference scores across our games (Tetris vs. Solitaire) using two-sided Bayesian *t* tests with the same prior as in the design analysis. This Bayesian approach lets us evaluate the strength of evidence in favor of either the H0 (no transfer of game play on mental rotation abilities) or the H1 (playing Tetris affects mental rotation abilities). We ran these Bayesian *t* tests for each mental rotation test individually because we expected potentially contradictory results between the different mental rotation tests based on previous research. Despite our main interest being concerned with the existence of an overall transfer effect of playing Tetris on mental rotation abilities, we ran three additional Bayesian ANOVAs including the factors game (Tetris vs. Solitaire) and gender (male vs. female) in order to investigate potential gender effects within our sample, as well as Bayesian ANCOVAs including expected outcomes regarding mental rotation abilities. We made the data publicly accessible on OSF (https://osf.io/d3wuc/).

## Results

We stopped recruiting new participants after we achieved the registered maximum sample size of at least 176 complete data sets for each game, because the data never satisfied the optional stopping rule for all three mental rotation tests (CRT, CCT, MRT). We then allowed all participants who had already started the study at this time point to complete it. Thus, 366 participants were included in the final sample (age: *M* = 25.52 years, *SD* = 5.69, range: 18–57), with 189 participants (120 female, 66 male, three nonbinary) that played Solitaire and 177 participants (113 female, 60 male, four nonbinary) that played Tetris.

## Confirmatory analyses

**Fig. 1:**
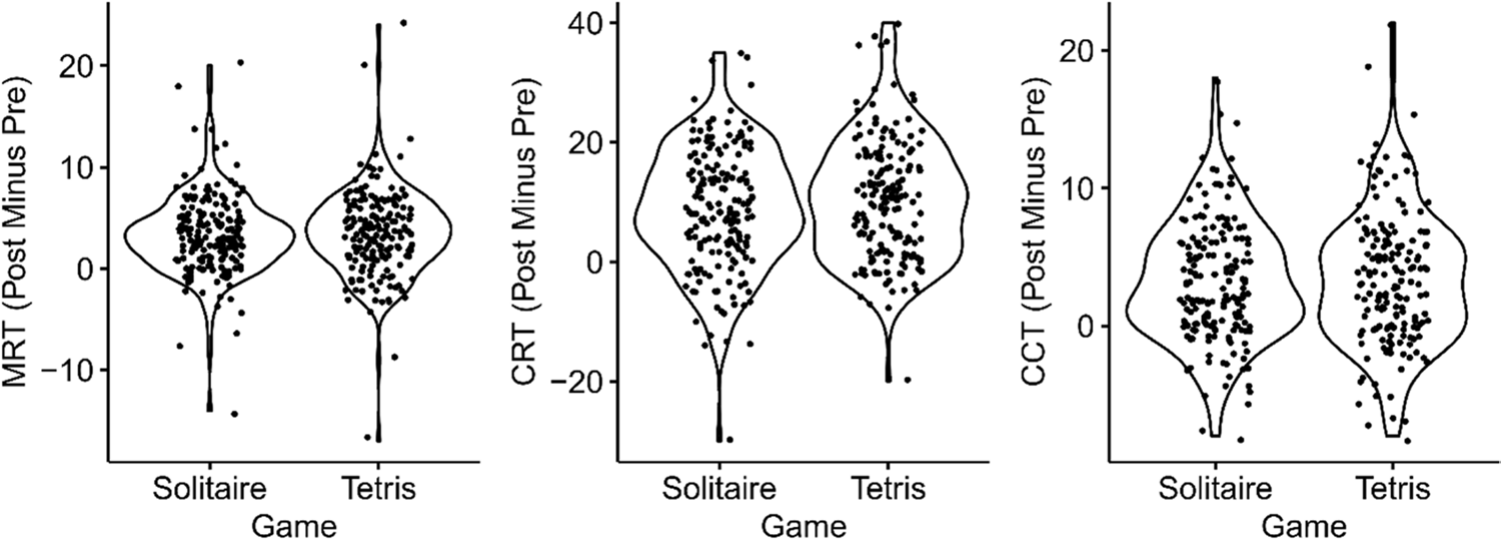
Violin plots of the pre–post difference scores. *Note.* Each dot represents the pre–post difference score of an individual participant (we added the dots with some jitter to the plot to improve the visibility of individual data points)

For the confirmatory (i.e., preregistered) analyses, we compared the pre–post difference scores for each mental rotation test (see Fig. 1)^2^ across our games (Tetris vs. Solitaire) using two-sided Bayesian *t* tests with the same prior as in the design analysis (performed with R Version 4.3.0 and the BayesFactor package Version 0.9.12-4.4). For the MRT (BF_10_ = 0.12) and the CCT (BF_10_ = 0.13), the observed Bayes factors crossed our preregistered Bayes factor boundaries (BF_10_ < 1/6 [≈ 0.167]) providing decisive evidence toward the null hypothesis, thus suggesting no specific transfer effect of playing Tetris on mental rotation abilities for both 3D mental rotation tests. For the CRT (BF_10_ = 0.37) the observed Bayes factor crossed none of the two preregistered Bayes factor boundaries (1/6 < BF_10_ < 10), thus providing an indecisive result for this 2D mental rotation test.

**Fig. 2:**
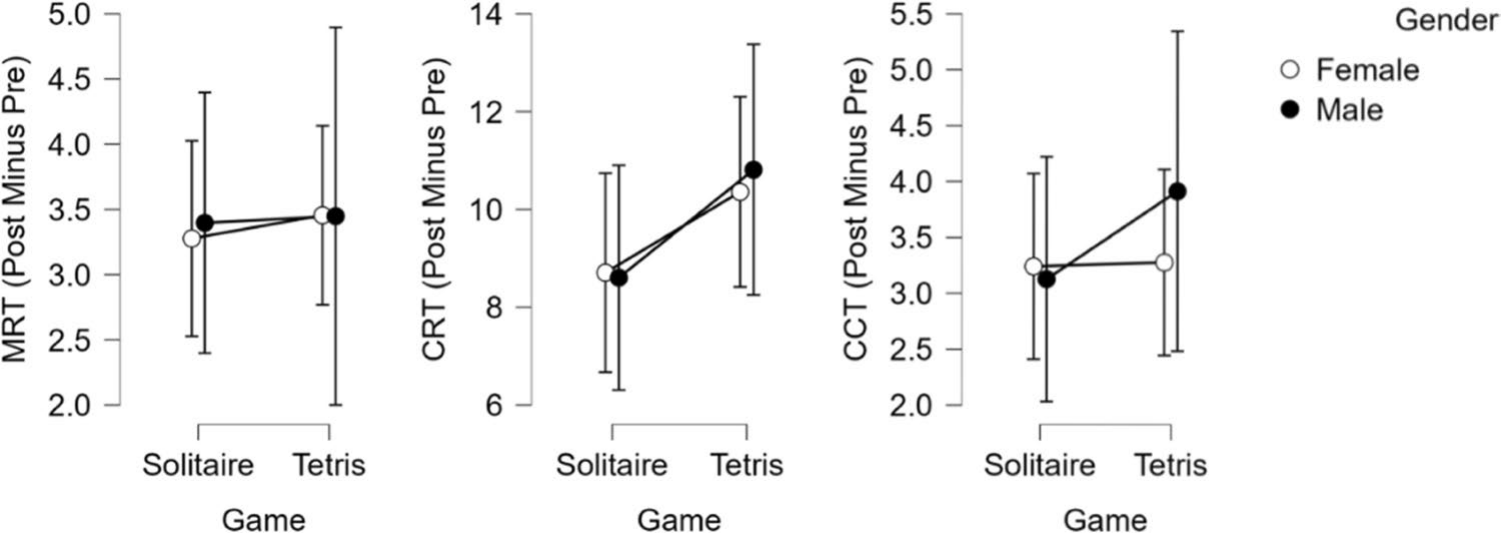
Pre–post difference scores separated by played game and gender of participants. *Note.* Error bars indicate 95% credible intervals

We performed the preregistered Bayesian ANOVAs and ANCOVAs (performed with JASP Version 0.17.2.1 with default parameters; analyses included all participants selecting male or female as gender) that indicated no influence of gender or expected outcomes on the pre–post difference scores in all three mental rotation tests (see Fig. 2 and Table 3).

**Table 3:** Analysis of effects for the Bayesian AN(C)OVAs

Mental Rotation Test (Analysis)	Effects	P(incl)	P(incl|data)	BF_inclusion_
MRT (ANOVA)	Game	0.600	0.112	0.084
	Gender	0.600	0.113	0.085
	Game × Gender	0.200	0.002	0.008
MRT (ANCOVA)	Game	0.600	0.112	0.084
	Gender	0.600	0.112	0.084
	Expected Outcome Tetris	0.500	0.130	0.150
	Expected Outcome Solitaire	0.500	0.128	0.147
	Game × Gender	0.200	0.002	0.008
CRT (ANOVA)	Game	0.600	0.319	0.313
	Gender	0.600	0.117	0.088
	Game × Gender	0.200	0.008	0.031
CRT (ANCOVA)	Game	0.600	0.318	0.311
	Gender	0.600	0.115	0.087
	Expected Outcome Tetris	0.500	0.115	0.130
	Expected Outcome Solitaire	0.500	0.191	0.237
	Game × Gender	0.200	0.006	0.025
CCT (ANOVA)	Game	0.600	0.126	0.096
	Gender	0.600	0.124	0.095
	Game × Gender	0.200	0.003	0.013
CCT (ANCOVA)	Game	0.600	0.123	0.094
	Gender	0.600	0.125	0.095
	Expected Outcome Tetris	0.500	0.352	0.544
	Expected Outcome Solitaire	0.500	0.139	0.161
	Game × Gender	0.200	0.003	0.014

## Exploratory analyses

We performed three exploratory Bayesian one-sample *t* tests of the pre–post difference scores against zero. Those tests provided strong evidence that there was an overall improvement in test scores from pretest to posttest for all three mental rotation tests: MRT (BF_10_ = 3.77 × 10^37^; *M*_Post-Pre_ = 3.39, *SE*_Post-Pre_ = 0.22), CRT (BF_10_ = 5.58 × 10^46^; *M*_Post-Pre_ = 9.54, *SE*_Post-Pre_ = 0.54), and CCT (BF_10_ = 5.64 × 10^29^; *M*_Post-Pre_ = 3.27, *SE*_Post-Pre_ = 0.25). Thus, the participants improved in their mental rotation test scores across the experiment, but this improvement did not depend on the game they played (see Confirmatory analyses).

We performed an exploratory two-sided paired-samples Bayesian *t* test comparing participants’ expected outcome of playing Tetris on mental rotation abilities with participants’ expected outcome of playing Solitaire on mental rotation abilities. This test provided strong evidence (BF_10_ = 2.35 × 10^64^) that participants expected a stronger impact of playing Tetris on mental rotation abilities (*M* = 1.60, *SE* = 0.04; ratings on 4-point scale: 1 = “auf jeden Fall” [English translation: “in any case”], 2 = “eher ja” [“rather yes”], 3 = “eher nein” [“rather no”], 4 = “auf keinen Fall” [“in no case”]) than playing Solitaire on mental rotation abilities (*M* = 2.94, *SE* = 0.05). We ran two additional exploratory two-sided Bayesian *t* tests to investigate whether participants’ expected outcome of playing Tetris or Solitaire on mental rotation abilities differed between our two experimental groups. As could be expected based on the random assignment of participants to the two experimental groups, this exploratory analysis suggested that the participants in our two experimental groups did not differ regarding their expectations of playing Tetris (BF_10_ = 0.29) or Solitaire (BF_10_ = 0.13) on mental rotation abilities.

**Fig. 3:**
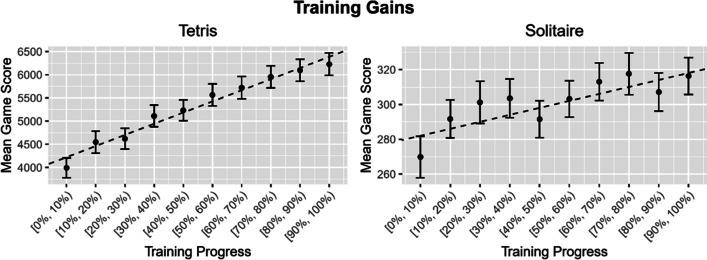
Training gains for Tetris and Solitaire. *Note.* The figure depicts the average game score of completed game sessions across training progress. The dashed line represents the median intercept and slope derived from the posterior distribution. The error bars indicate the standard error of the mean. Training progress intervals are marked with the brackets [X, Y). The opening bracket “[” indicates the starting point of the interval, while the closing bracket “)” indicates that the point is almost reached

One potential concern regarding the evidence against transfer effects in our study is whether participants showed any training gains at all. Without participants improving performance within the played game during training, it is not clear why one would expect transfer. Thus, we performed an exploratory analysis on the development of participants’ mean game scores across training (see Fig. 3). This analysis included the average game score of completed game sessions, thus excluding sessions terminated due to participant inactivity, across training progress (0–100% in steps of 10%). The last game was omitted for this analysis. For Tetris and Solitaire separately, we fitted Bayesian mixed models with training progress as fixed effect and the intercept of participant as random effect and compared those models against models omitting training progress as fixed effect. This provided evidence in favor of the inclusion of training progress as fixed effect (Tetris: BF_10_ = 3.24 × 10^64^, Solitaire: BF_10_ = 11744.77), indicating that participants improved their performance within the played game during training.

## Discussion

Transfer effects of video games on cognitive performance are controversially discussed (Bediou et al., 2018), and there might be no reliable far transfer effects of video game play (Sala et al., 2018). Given the contradictory findings regarding the existence of even short-term near transfer effects, such as playing Tetris on mental rotation abilities (see Table 2), we aimed at contributing to this discussion by the publication of a data set that is unbiased by its statistical significance. With our present study, we conducted a registered conceptual replication of the short-term transfer effect of playing Tetris on mental rotation abilities, which is one of the most used and cited short-term near transfer effects (e.g., Boot et al., 2008; De Lisi & Wolford, 2002; Okagaki & Frensch, 1994).

We observed decisive evidence for the null hypothesis for two mental rotation tests (MRT, CCT), thus providing evidence against the idea of a general short-term transfer effect of playing Tetris on mental rotation abilities. It is unlikely that this lack of transfer effect is due to training time being too short, as we were able to demonstrate substantial training gains over the training period, and our study used a training time of 10 hours in four weeks, which is in the range of training times used in previous studies (see Table 2). Despite the large sample size, the results regarding the third mental rotation test (CRT) remained indecisive. The evidence against transfer effects for the 3D mental rotation tests (MRT, CRT) matches with prior research suggesting that training with 2D Tetris does not transfer to 3D mental rotation (Moreau, 2013). We can only speculate regarding the indecisive result regarding the 2D mental rotation test (CRT). There could be a so far undiscovered moderator causing transfer effects for some participants but not for other participants, or this finding could also just be a chance finding, with future studies potentially providing more decisive evidence.

We observed reliable repeated testing effects for all three mental rotation tests. That is, participants reliably improved in their mental rotation test scores, both for playing Tetris and for playing Solitaire. This demonstrates the importance of having an active control group within experimental studies investigating transfer effects of video game play. Drawing inference based on sole pre–post difference scores without an appropriate control group could lead to falsely reporting transfer effects despite the existence of mere repeated testing effects.

Prior research studying transfer effects of playing Tetris on mental rotation skills also focused on the influence of gender. While some studies suggested that the transfer effect is stronger for one gender (Cherney, 2008; De Lisi & Cammarano, 1996), other studies found no transfer effect differences between genders or mixed findings (De Lisi & Wolford, 2002; Okagaki & Frensch, 1994; Terlecki et al., 2008). Our present study provides further evidence against the influence of gender on the improvement in mental rotation test scores, neither on the repeated testing effect nor any Tetris-specific (nonexistent) transfer effect.

In summary, we investigated the frequently cited and controversially discussed short-term transfer effect of playing Tetris on mental rotation abilities, employing three common mental rotation tests. Our results speak against such a transfer effect and also against a gender-specific influence.

## Footnotes

1 In order to mask our interest in those two particular items, we measured them within a questionnaire asking about the expected influence of four games (Tetris, Solitaire, FIFA, Grand Theft Auto [GTA]) regarding four abilities [mental rotation, memory performance, visual attention, calculation] on a rating scale ranging from 1 (*strongly agree*) to 4 (*strongly disagree*) with a random order of questions for each participant.

2 After the completion of data collection, we noticed that due to an error in the experiment, the duration of a playing session was logged as being too long in very rare cases. This resulted in some participants being able to continue to the posttest before they completed the intended playing time. The best proxy for actual playing time of participants is the sum of all game durations excluding the last game. To remove participants that played far too less (11 × Solitaire, 3 × Tetris), we only kept those participants with at least 8 hours of play before the start of the last game. For the remaining participants the average playing time was about the intended 10 hours (mean playing time before the start of the last game across all remaining participants: *M*_Tetris_ = 9.88 hours, *M*_Solitaire_ = 9.91 hours). For a low number of participants (8 × Solitaire, 5 × Tetris), actual playing time was slightly above 10 hours (mostly a few minutes, with most extreme case being 1.5 hours). Within the results section, we report the statistical values of the data set containing all participants with at least 8 hours of play time before the last game. Note that we obtained the same qualitative conclusions when performing the analyses with either the whole data set or a data set that additionally excludes the participants with too-long playing times.
